# Circadian Regulation of Glutathione Levels and Biosynthesis in *Drosophila melanogaster*

**DOI:** 10.1371/journal.pone.0050454

**Published:** 2012-11-30

**Authors:** Laura M. Beaver, Vladimir I. Klichko, Eileen S. Chow, Joanna Kotwica-Rolinska, Marisa Williamson, William C. Orr, Svetlana N. Radyuk, Jadwiga M. Giebultowicz

**Affiliations:** 1 Department of Zoology, Oregon State University, Corvallis, Oregon, United States of America; 2 Department of Biological Sciences, Southern Methodist University, Dallas, Texas, United States of America; 3 Department of Animal Physiology, Zoological Institute, University of Warsaw, Warsaw, Poland; Karlsruhe Institute of Technology, Germany

## Abstract

Circadian clocks generate daily rhythms in neuronal, physiological, and metabolic functions. Previous studies in mammals reported daily fluctuations in levels of the major endogenous antioxidant, glutathione (GSH), but the molecular mechanisms that govern such fluctuations remained unknown. To address this question, we used the model species *Drosophila*, which has a rich arsenal of genetic tools. Previously, we showed that loss of the circadian clock increased oxidative damage and caused neurodegenerative changes in the brain, while enhanced GSH production in neuronal tissue conferred beneficial effects on fly survivorship under normal and stress conditions. In the current study we report that the GSH concentrations in fly heads fluctuate in a circadian clock-dependent manner. We further demonstrate a rhythm in activity of glutamate cysteine ligase (GCL), the rate-limiting enzyme in glutathione biosynthesis. Significant rhythms were also observed for mRNA levels of genes encoding the catalytic (*Gclc*) and modulatory (*Gclm*) subunits comprising the GCL holoenzyme. Furthermore, we found that the expression of a glutathione S-transferase, *GstD1*, which utilizes GSH in cellular detoxification, significantly fluctuated during the circadian day. To directly address the role of the clock in regulating GSH-related rhythms, the expression levels of the GCL subunits and *GstD1*, as well as GCL activity and GSH production were evaluated in flies with a null mutation in the clock genes *cycle* and *period*. The rhythms observed in control flies were not evident in the clock mutants, thus linking glutathione production and utilization to the circadian system. Together, these data suggest that the circadian system modulates pathways involved in production and utilization of glutathione.

## Introduction

Circadian clocks generate a multitude of circadian rhythms in behavioral, neuronal, physiological, and endocrine functions [1], [2]. While these rhythms have endogenous periodicity of circa 24 h, in nature they are entrained by light and temperature cycles associated with solar days. Circadian clocks consist of transcriptional and translational feedback loops working in a cell autonomous manner that are largely conserved between *Drosophila* and humans [3], [4]. At the core of the *Drosophila* circadian clock there are four clock genes: *Clock* (*Clk*), *cycle* (*cyc*), *timeless* (*tim*), and *period* (*per*) [5]. They interact in a negative feedback loop, such that loss of function in any of these genes results in disruption of the clock mechanism [6]. The expression levels of *per* and *tim* are regulated by transcriptional activators encoded by *Clk* and *cyc*. This leads to periodic increase in the levels of PER and TIM proteins. The latter accumulate in cell nuclei, and repress CLK/CYC activators, leading to suppression of *per* and *tim* transcription. In addition to *per* and *tim*, CLK/ CYC heterodimers activate genes that participate in additional clock feedback loops and a substantial number of clock output genes [7], [8]. Clock-controlled output genes modulate a myriad of metabolic and cellular functions, such as the regulation of energy balance, DNA-damage repair and xenobiotic detoxification in both mammals [9]–[11] and *Drosophila* [12]–[14].

There is emerging evidence that circadian clocks regulate processes that protect an organism from oxidative stress. Previously, we reported that levels of reactive oxygen species (ROS) and protein carbonyls fluctuate in a daily rhythm in heads of young wild type flies, whereas they were non-rhythmic and significantly higher in clock deficient *per^01^* mutants [15]. These mutants also accumulated higher levels of protein carbonyls and peroxidated lipids during aging [16], [17], suggesting that antioxidant defenses were compromised by the loss of clock function. In mice, deficiency of the clock protein BMAL1 (homolog of fly CYC protein) leads to increased ROS levels in several tissues [18]. However, it is not understood which pathways involved in protecting cells from oxidative stress may be modulated by the circadian system.

To combat oxidative stress and minimize the accumulation of oxidative damage, organisms developed a complex network of antioxidant defenses, capable of ROS removal. Glutathione (GSH) is a central player in this network, able to protect cells from oxidative stress, regulate activity of detoxification enzymes and mediate redox-sensitive signaling [19], [20]. Previous studies reported daily changes in GSH levels in different mammalian organs [21] but the role of circadian mechanism in these fluctuations has not been addressed. Genome-wide analyses of circadian transcriptome in fly heads by microarray [8], [22]–[24], or RNA-seq [25] suggests that the expression of some genes comprising glutathione-synthesizing and conjugating systems may occur in a circadian manner. Here we utilized the genetic tools available in *Drosophila* to determine whether there is a causal relationship between circadian clocks and GSH-related pathways. We uncovered daily oscillations in glutathione levels in fly heads and investigated the molecular mechanisms underlying this rhythm. We report that the circadian clock is involved in regulating *de novo* glutathione biosynthesis.

## Methods

### Fly rearing and strains

*Drosophila melanogaster* were raised on yeast (35 g/L), cornmeal and molasses diet at 25±1°C, at low density to attain uniform size, under a 12 h light/12 h dark (LD) regimen (where Zeitgeber time (ZT) 0 is time of lights on and ZT 12 is time of lights off). Flies were separated 1–2 days after emergence, and five day old males were used for all experiments. For constant darkness (DD) experiments, flies were collected on the second day of DD where the time when lights would have turned on is designated by CT 0 and off is CT 12. Clock mutants *cyc^01^* [26] and *per^01^* [27] were backcrossed to the Canton S (CS) control line allowing free recombination for at least 8 generations to isogenize the genetic background. All work was completed on heads that were isolated following freezing using a dry ice and sieve method.

### Quantitative real-time PCR

Male heads were homogenized in TriReagent following manufacturer's protocol (Sigma-Aldrich Co., St. Louis, MO) using a Kontes handheld motor. Samples were purified using the RNeasy mini kit (Qiagen, Valencia, CA) with on-column DNAse digestion (Qiagen) or by rDNAse I (Takara, Otsu, Shiga, Japan) followed by sodium acetate precipitation. Synthesis of cDNA was achieved with iScript cDNA synthesis kit (Bio-Rad, Hercules, CA) according to manufacturer's protocol. Real-time PCR was performed with iTaq SYBR Green Supermix with Rox (Bio-Rad) on an ABI Step-One Plus real-time machine. Primers were obtained from IDT (Coralville, IA). All primers used in this study had efficiency >96%, and their sequences are shown in Table S1. Data were normalized to the gene *rp49* or *robl* [28] as indicated in the results and analyzed using the standard 2^−ΔΔCT^ method.

### Immunoblot analysis

Samples were collected at 4 h intervals from 15–20 whole heads obtained from CS flies and processed as described [29]. Briefly, proteins were extracted from heads, and ∼5 µg of protein was resolved by PAGE for each sample. Immunoblots were performed with antibodies generated against recombinant GCLc and GCLm proteins [29] and anti-actin antibodies (MP Biomedicals, Santa Anna, CA) to control for loading. The intensity of signals was analyzed by densitometric scanning, using the digital imaging analysis system with AlphaEase Stand Alone Software (Alpha Innotech Corp., San Leandro, CA). Signals were standardized against the signals obtained for actin or against the densitometry of Coomassie staining.

### Glutamate cysteine ligase (GCL) enzyme activity and glutathione levels

GCL enzyme activity was measured as described [30] with some modifications (see Supplementary Methods S1). Briefly, ∼50 fly heads were homogenized in 0.3 ml extraction buffer (320 mM sucrose, 1mM PMSF, 1 mM 6-aminohexanoic acid, 10 mM Tris, pH 7.4) and centrifuged at 14000 g for 5 min at 4°C. Low-molecular weight components were removed from the supernatant by ultrafiltration at 14000 g for 10 min at 4°C using Amicon Ultra Centrifugal Filter with a 10 kDa cut-off (EMD Millipore Corp., Billerica, MA). The protein preparations were washed with 0.3 ml buffer (200 mM sucrose, 1 mM PMSF, 1 mM 6-aminohexanoic acid, 10 mM Tris, pH 7.4) by additional ultrafiltration. Protein concentrations were determined using the DC Bio-Rad protein assay (Bio-Rad). GCL activity reaction was performed immediately after protein preparation, as suggested by [31]. The reaction was initiated by mixing 20–35 µg protein with a reaction mixture containing 10 mM ATP, 5 mM L-cysteine, 50 mM L-glutamate, 500 µM acivicin, 20 mM MgCl_2_, 100 mM Tris-HCl pH 8.2 in a total volume of 150 µl followed by incubation for 15 min at 25°C. Each reaction was duplicated. Time, protein and substrate concentration linearity were determined in pilot experiments. The specificity of the assay was tested using the GCL inhibitor L-buthionine-S, R-sulfoximine. The reaction was terminated by adding an equal volume of freshly prepared 10% (w/v) meta-phosphoric acid (MPA) containing 10 µM L-methionine as internal standard for HPLC analysis. Precipitated proteins were removed by centrifugation and the supernatant was filtered through 0.22 µm PTFE membrane syringe filter HPLC/GC quality (Phenomenex, Inc., Torrance, CA). Filtrates were immediately analyzed by HPLC or stored at −80°C for no longer than 24 h before analysis.

GSH content in the head homogenates was quantified by HPLC as described [30] with some modifications (see Supplementary Methods S1). Briefly, 50 heads were homogenized in 200 µl of freshly prepared ice-cold 5% MPA. After 30 min incubation on ice and centrifugation at 16000 g for 20 min at 4°C, the amount of protein in the precipitate was determined using the DC protein assay (Bio-Rad Laboratories) according to manufacturer's recommendations (Bulletin 1770 US/EG Rev A, Bio-Rad). The supernatant was divided into two aliquots. The first aliquot was neutralized with 1N NaOH, treated with 2 mM N-ethylmaleimide for 30 sec on ice, and terminated with an equal volume of 10% MPA. The second aliquot was adjusted to the final volume of the first aliquot with 5% MPA. Samples were then filtered through 0.22 µm PTFE membrane syringe filter and immediately analyzed by HPLC or stored at −80°C.

Samples from the GCL assay and the free aminothiol extractions were analyzed by HPLC equipped with the Agilent 1100 quaternary pump, degasser, thermostated autosampler, and column compartment (Agilent Technologies, Germany). The separation was performed under isocratic elution using a reverse-phase C18 Gemini-NX (3 µm, 4.6×150 mm) column (Phenomenex, Torrance, CA) with the flow rate of 0.75 ml/min. The mobile phase contains 2% (v/v) acetonitrile, 25 mM monobasic sodium phosphate, 0.5 mM 1-octane sulfonic acid as ion-pairing agent, pH 7.2, adjusted with ortho-phosphoric acid. Following separation, aminothiols were detected using the 5600 CoulArray electrochemical detector equipped with four-channel analytical cell (ESA, Inc., Chelmsford, MA). For measuring γ-GC, we used increasing potentials (+100, +200, +750, +850 mV in channels 1–4, respectively). γ-GC and L-methionine were detected in channel 3 at +750 mV. Calibration standards containing 1, 2, 3 and 4 µM γ-GC (Sigma-Aldrich) were prepared in 5% (w/v) MPA containing 5 µM L-methionine (Sigma-Aldrich) as an internal standard and injected at regular intervals. Peak areas normalized to an internal standard were used for determining concentrations of γ-GC.

Potentials of +400, +600, +750, and +875 mV were used for GSH detection. GSH was detected in channel 3 at +750 mV. Each sample was injected twice. GSH concentrations were calculated as differences between peak areas corresponding to untreated and N-ethylmaleimide-treated aliquots of the sample. Calibration standards containing 0.1, 0.3, 1, 3, 10 and 30 µM GSH (Sigma-Aldrich) were prepared in 5% (w/v) MPA and injected at regular intervals.

## Results

### Circadian clock regulates GSH levels in fly heads

**Figure 1:**
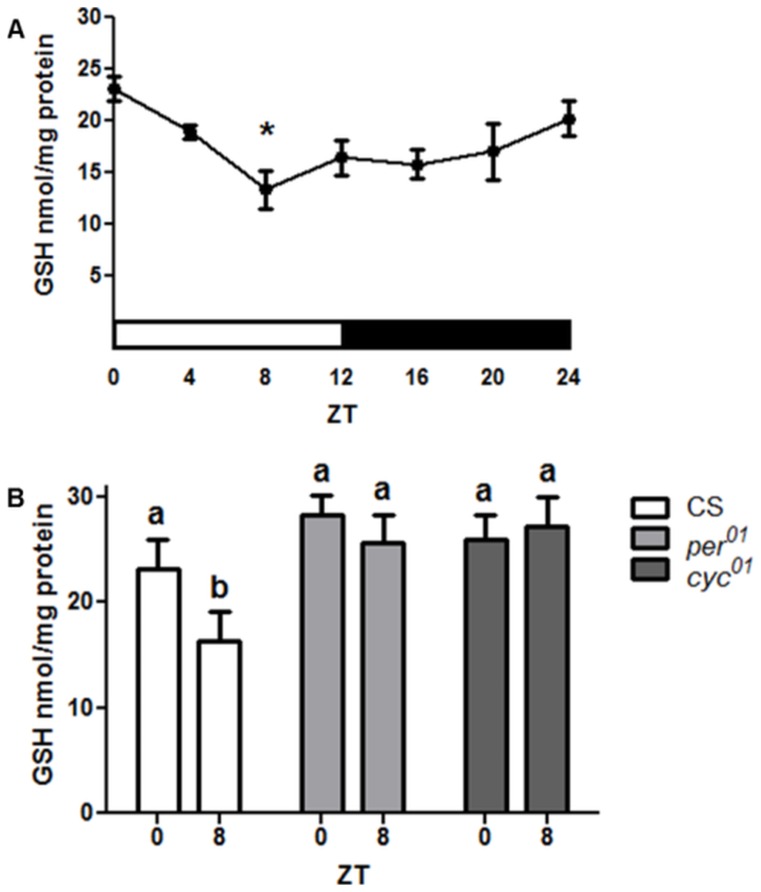
Circadian regulation of GSH levels in *Drosophila* heads. (A) Daily changes in GSH levels in wild type CS males. Data represents average values ± SEM obtained from 4 independent bio-replicates (total N = 8). Data were analyzed by a 1-way ANOVA and Bonferroni's post-tests where an asterisk marks significantly lower values relative to ZT 0 (p<0.05). White horizontal bar marks the time when light is on; black bar denotes darkness. (B) GSH levels were altered in *per^01^* and *cyc^01^* mutants such that no statistical difference was detected between time points where control CS flies showed a peak (ZT 0) and a trough (ZT 8). Bars represent average values ± SEM obtained from 3–4 independent bio-replicates (± SEM). Data in (B) were analyzed by a 2-way ANOVA and Bonferroni's post-tests. Different subscript letters indicate significant difference between treatment groups. ZT = Zeitgeber Time.

We measured GSH levels in heads of wild type Canton S (CS) control flies collected at 4 h intervals around the clock and found significant oscillations with 1.5-fold amplitude such that the highest GSH concentrations were detected in the early morning at ZT 0 followed by a decline to a trough in midday at ZT 8 (Fig. 1A). To test whether the GSH rhythm is controlled by the clock mechanism, we measured GSH in heads of arrhythmic clock mutants with loss of *cyc* (*cyc^01^*) or *per* (*per^01^*) function. In contrast to control CS flies, no significant difference between peak and trough times was found in *per^01^* or *cyc^01^* mutants (Fig. 1B). Furthermore, the trough in levels of GSH observed in the control was absent in the arrhythmic mutants.

### Expression of genes involved in glutathione synthesis is modulated by the circadian clock

**Figure 2:**
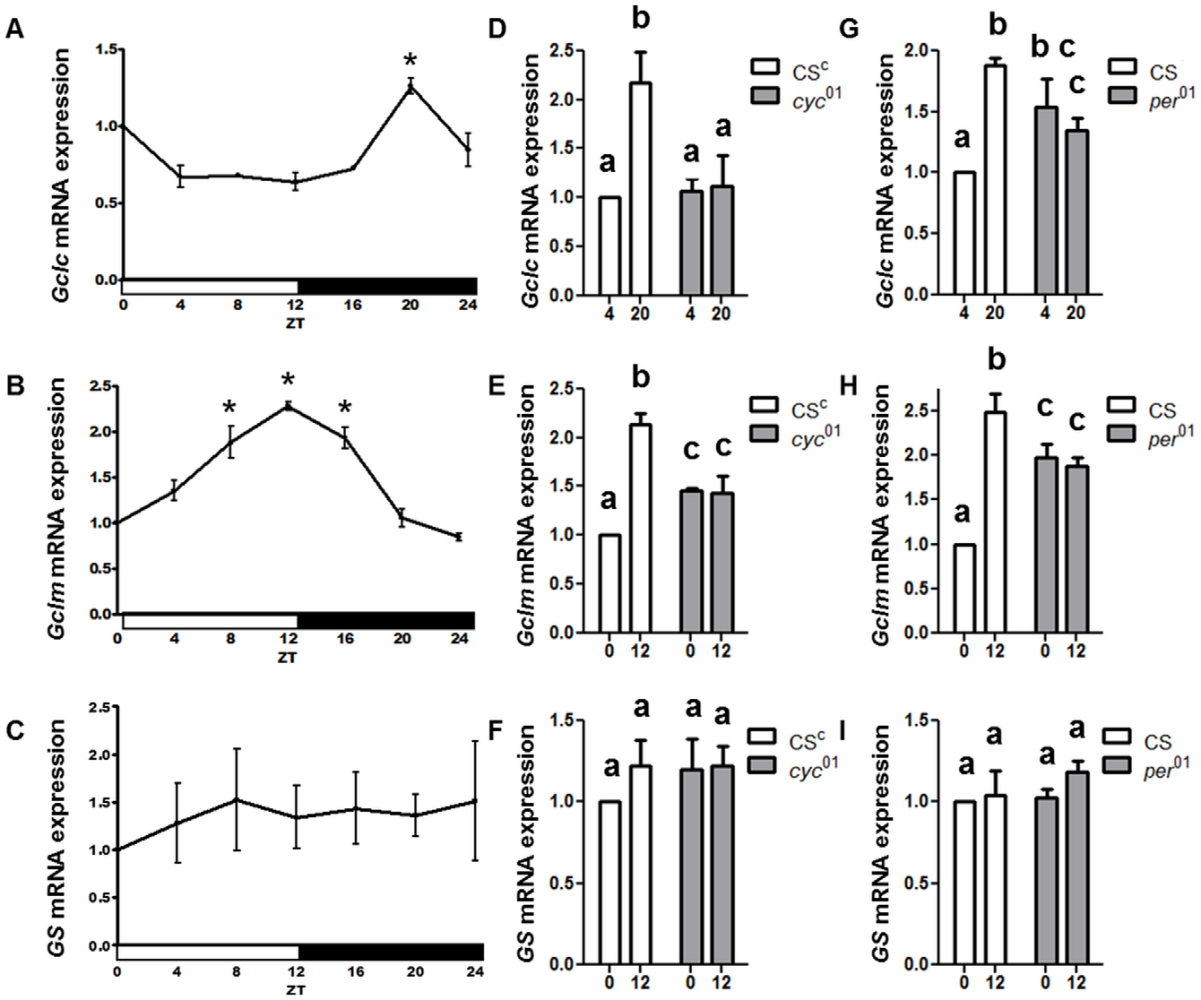
Circadian regulation of *Gclc* and *Gclm* mRNA expression levels in fly heads. There is a significant rhythm in *Gclc* (A) and *Gclm* (B) mRNA but not in *GS* mRNA profile (C). Data for (A–C) were analyzed by a 1-way ANOVA and Bonferroni's post-tests, and an asterisk marks significantly higher values relative to the lowest value (p<0.05). In *cyc^01^* mutants, the peak in *Gclc* (D) and *Gclm* (E) is abolished while *GS* mRNA is not affected (F). In *per^01^* mutants, the trough-to-peak differences in *Gclc* (G) and *Gclm* (H) are abolished while *GS* is not changed (I). Different subscript letters in (D–I) indicate a significant difference between treatment groups. All graphs are average values obtained from 3–5 independent bio-replicates (± SEM) and normalized to ZT 0 or ZT 4 as appropriate.

Given the rhythmic fluctuations in GSH levels, we investigated the daily profiles of the genes involved in GSH biosynthesis. Genes encoding the catalytic (*Gclc*) and modulatory (*Gclm*) subunits of the rate-limiting GCL holoenzyme were examined. We also examined the gene encoding second enzymatic step, glutathione synthase (*GS*). Analysis of the mRNA revealed daily oscillations in the expression of *Gclc* and *Gclm* in LD (Fig. 2A and 2B), while no significant diurnal fluctuations were found in the *GS* mRNA levels (Fig. 2C). The levels of both *Gclc* and *Gclm* mRNA oscillated in a rhythmic fashion with a significant, about two-fold amplitude between the peak and trough time points. Interestingly while a sharp peak of *Gclc* mRNA was detected at late night (ZT 20), the peak of *Gclm* expression was much broader (ZT 8–16) and phase advanced relative to the *Gclc* peak (Fig. 2A–B). To determine whether the expression of *Gclc* and *Gclm* was regulated by the circadian clock, mRNA levels were examined in *per^01^* and *cyc^01^* mutants at times when wild type flies showed trough and peak expression levels for each gene. In *cyc^01^, Gclc* mRNA levels were significantly lower at the time point when control flies showed peak expression (Fig. 2D). In contrast, in the *per^01^* flies, *Gclc* mRNA levels were significantly higher as compared to the trough time in control flies (Fig. 2G). With regard to *Gclm*, mRNA levels were intermediate in both clock deficient genotypes, that is, significantly higher in *per^01^* and *cyc^01^* than control flies the trough time point, but significantly lower at ZT 12, the peak time point (Fig. 2E and 2H). *GS* mRNA levels were not altered in *cyc^01^* or *per^01^* flies (Fig. 2F and 2I).

**Figure 3:**
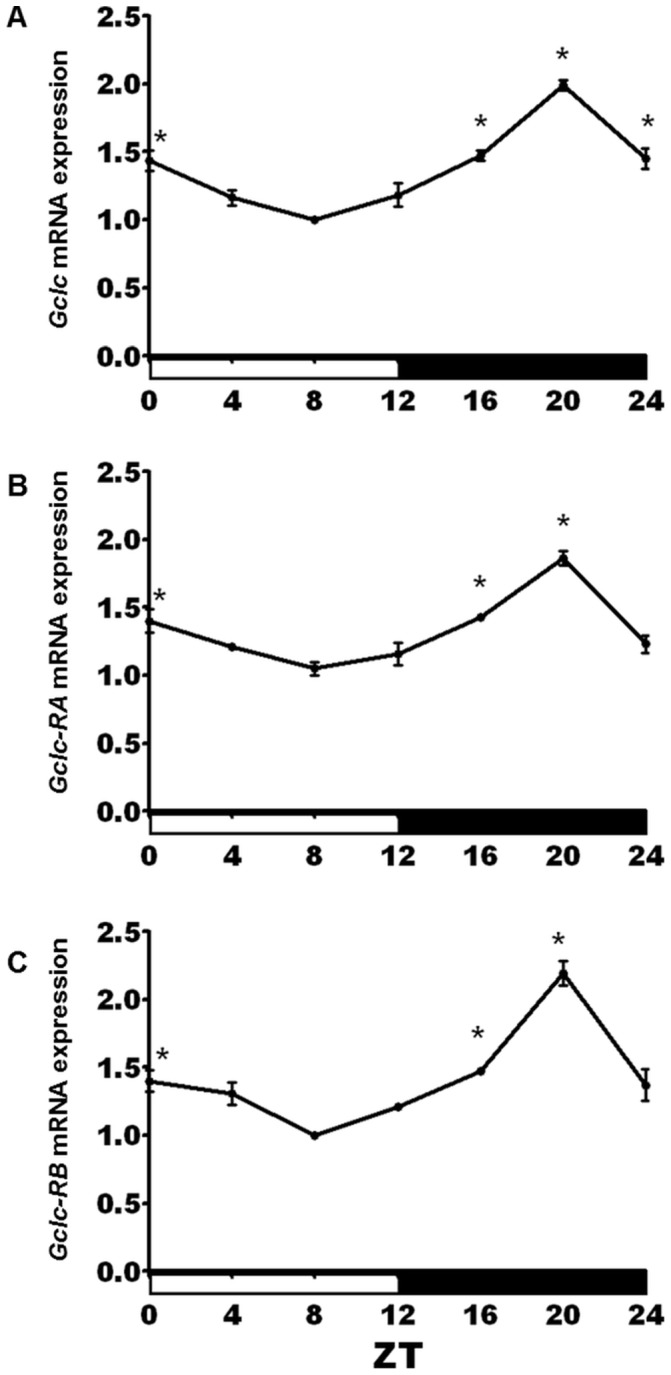
Circadian expression of GCLc isoforms. Daily oscillations in (A) total *Gclc* mRNA levels were also significant when (B) *Gclc-RA* and (C) *Gclc-RB* isoforms were measured separately using isoform-specific primers. The two isoforms share the same coding regions, but have distinct 5′ UTR regions. All graphs are average values obtained from 3 independent bio-repeats each normalized to the time point with the lowest expression. An asterisk indicates significant difference from the trough based on a 1-way ANOVA and Bonferroni's post-tests (p<0.05).

The observed expression levels of *Gclc* and *Gclm* in *per^01^* flies (lack of a trough in the morning) and in *cyc^01^* flies (lack of a peak in the evening) suggest that transcription of both genes is positively regulated by the CYC/CLK protein complex and negatively regulated by the PER protein. Transcriptional activation of *Gclc* and *Gclm* by CLK/CYC would be consistent with the recent genome-wide ChIP-chip study showing that CLK/CYC complexes are bound in the vicinity of *Gclc* and *Gclm* promoters in a time-dependent manner [7]. However, in both cases, CLK binding occurred near another transcription start site on the opposite DNA strand. Thus, these alternate genes, *CG1575* and *CG17625*, could have been the CLK/CYC targets instead of, or in addition to, *Gclc* and *Gclm*. To explore this issue, we conducted qRT-PCR studies. We determined that *CG17625* is not expressed in adult heads, consistent with fly atlas data [32] and that *CG1575*, which is adjacent to *Gclc*, did not display rhythms consistent with CLK targets (data not shown). As the *Gclc* gene encodes two isoforms, RA and RB, that share the same coding regions but have distinct 5′ UTR regions [33], [34], we determined the daily profile of both transcripts, using subunit-specific primers. Data revealed that both isoforms have rhythmic expression with a significant peak at ZT 20 (Fig. 3). Previous studies showed that deletion of the 5′ UTR associated with the RA transcript results in lethality [34], suggesting that the *Gclc*-RA isoform is essential for survival.

**Figure 4:**
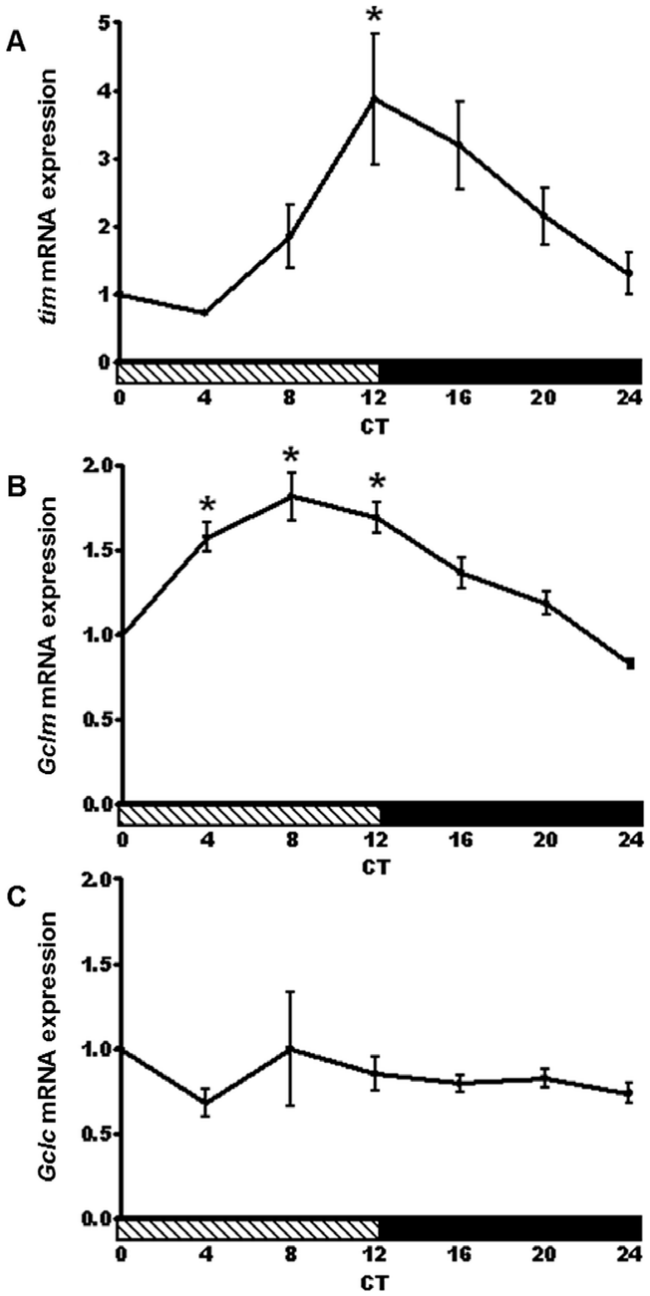
Circadian rhythm in *Gclm* expression persists in constant darkness. (A) *tim* and (B) *Gclm* mRNA expression show a circadian rhythm in heads of CS flies on the second day of constant darkness. An asterisk indicates a significant difference in the expression level between the trough of each gene and the peak (p<0.05). (C) No significant rhythm was detected in *Gclc* mRNA levels in wild type flies. Data represents average values obtained from 3 independent bio-replicates (± SEM) and normalized to ZT 0. Significance was calculated by a 1-way ANOVA and Bonferroni's multiple comparison post-tests. CT = Circadian Time. Shaded horizontal bars indicate subjective day.

A key feature of the circadian clock is that rhythmic variations in the mRNA levels of clock genes such as *tim* are maintained under constant darkness (DD) [5]. Our qRT-PCR analysis of head samples isolated from flies kept in DD revealed that *tim* maintained a 4-fold mRNA amplitude between CT 4 and CT 12 (Fig. 4A) on the second day in DD. In addition, a significant circadian rhythm in *Gclm* mRNA levels was evident in DD (Fig. 4B). On the other hand, the *Gclc* mRNA rhythm was not sustained in DD (Fig. 4C).

### GCL enzyme activity displays a daily rhythm which is abolished in clock mutants

**Figure 5:**
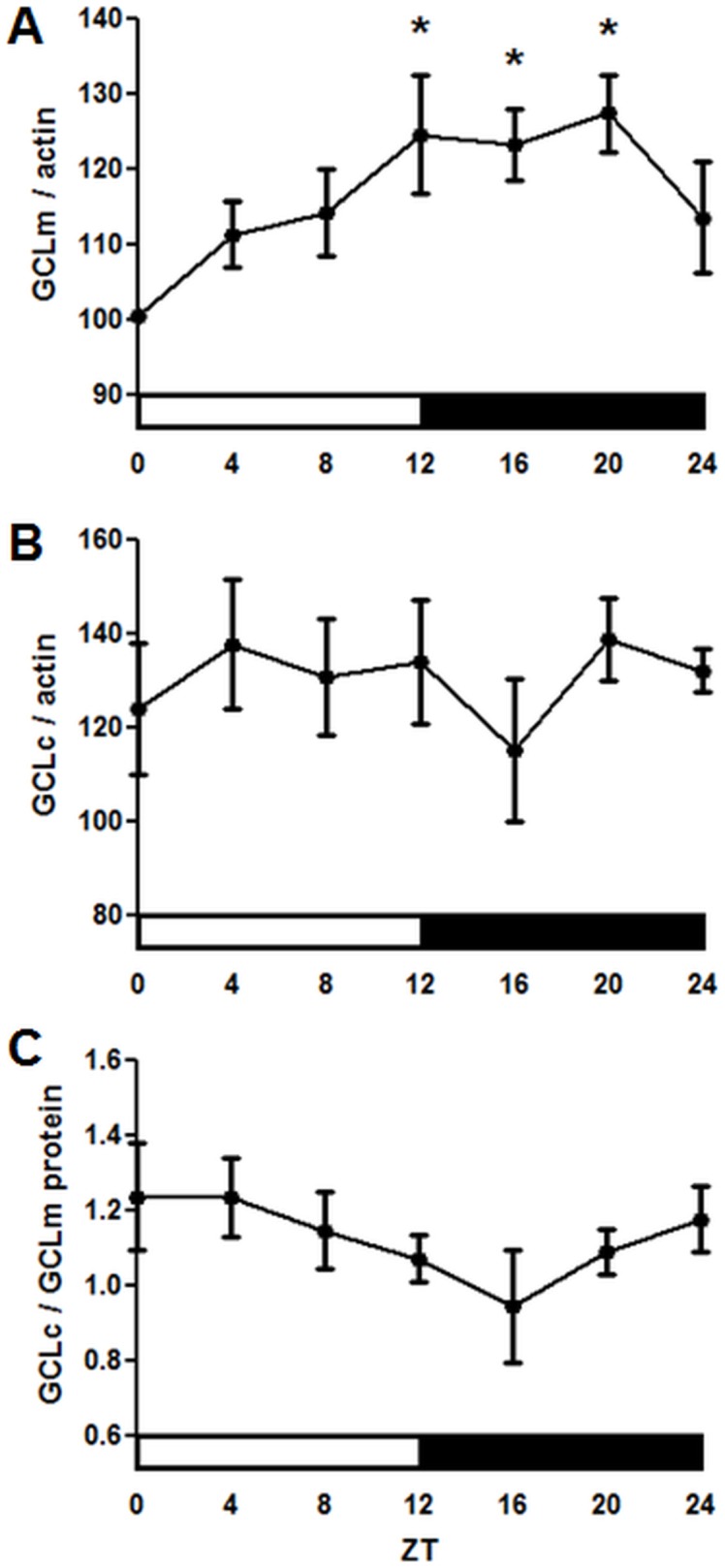
Profiles of GCL proteins and their ratio over the circadian day in the heads of wild type CS males. (A) GCLm and (B) GCLc protein levels based on average densitometry of signals obtained on Western blots with anti-GCLc or anti-GCLm antibodies normalized to signals obtained with anti-actin antibodies. Each replicate was normalized to the time point with the lowest expression. (C) Ratio of GCLc to GCLm protein over the circadian day in wild type CS males. (A–C) Data represent average values ± SEM obtained from 8 immunoblots performed with 4 independent bio-replicates. Statistical significance was determined by a 1-way ANOVA and Dunnett's post-test as denoted by asterisks (p<0.05).

**Figure 6:**
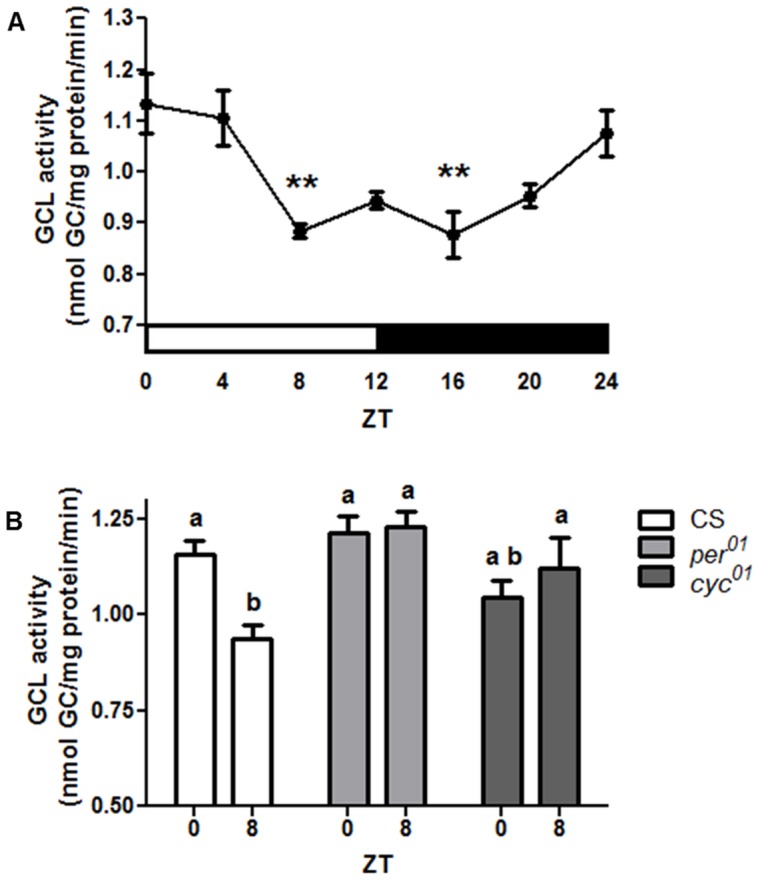
Circadian regulation of GCL enzymatic activity. (A) Daily profile of GCL activity in heads of CS flies as measured by the formation of the GCL product, *γ*-GC. Data represents average values ± SEM obtained from 4 independent bio-replicates (total N = 16). An asterisk indicates a significant difference between the peak and trough time points calculated by 1-way ANOVA and Bonferroni post-tests. (B) GCL activity was altered in *per^01^* and *cyc^01^* mutants such that no statistical difference was detected between time points where control CS flies showed peak at (ZT 0) and trough (ZT 8). Bars show average values ± SEM obtained from 4–5 independent bio-replicates (total N = 16). Data in (B) are analyzed by 2-way ANOVA and Bonferroni's post-tests. Different subscript letters indicate significant differences between treatment groups (p<0.05).

In contrast to *Gclm* mRNA levels, Western blot analysis showed less pronounced but significant changes in GCLm protein levels with a trough at lights on (ZT 0) and a peak in the evening (ZT 12–20) (Fig. 5A). GCLc protein did not show significant changes during the 24 h period (Fig. 5B). It is known that the catalytic activity of the GCL holoenzyme is regulated at different levels and depends on many factors, including interactions between GCLc and GCLm, as well as their concentrations and molar proportions [35]–[37]. Although only minor variations in the protein levels of individual GCL subunits were observed, the ratios of GCLc to GCLm changed by about 40% around the clock (Fig. 5C), and this could affect GCL catalytic activity. Importantly, we found significant changes in GCL activity in wild type flies throughout the circadian day. The peak in GCL activity was in the morning at ZT 0–4, and the lowest activity was between ZT 8–16 (Fig. 6A). Next, we examined whether GCL activity was altered in the *per^01^* or *cyc^01^* mutants. Neither mutant showed a significant difference in GCL activity at the time points when control (CS) flies showed a peak (ZT 0) and trough (ZT 8, Fig. 6B). Thus, we determined that the circadian clock regulates glutathione synthesis by affecting activity of the GCL holoenzyme.

### Circadian regulation of GstD1 expression

**Figure 7:**
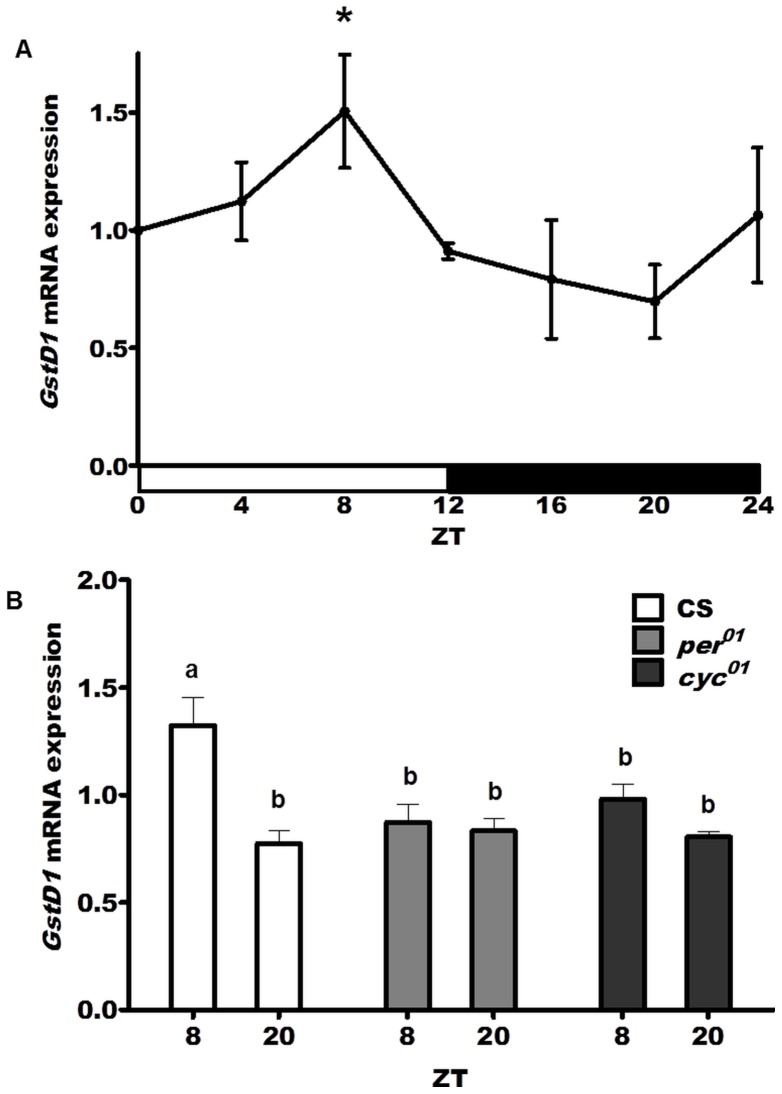
Circadian regulation of *GstD1* expression. (A) A circadian rhythm in *GstD1* mRNA levels was detected in wild type (CS) flies with a peak at ZT 8 significantly different from the trough at ZT 20 (p<0.01). (B) No significant difference was observed between ZT 8 and ZT 20 in *per^01^* and *cyc^01^* flies while the difference was observed in CS heads (p<0.01). Data represent average values (± SEM) obtained from 3 independent bio-replicates and normalized to ZT 0. Data were analyzed by a 2-way ANOVA and Bonferroni's post-tests. Different subscript letters indicate significant difference between treatment groups.

One of the major defense functions of glutathione is the elimination of xenobiotics, as well as metabolic by-products, by conjugating these compounds to glutathione in reactions mediated by the family of enzymes designated as the glutathione transferases (GSTs). Given the decline in GSH levels in midday (Fig. 1), we examined mRNA levels of *glutathione S-transferase D1* (*GstD1*), a known antioxidant and detoxification response gene in *Drosophila* [38], [39]. We found a statistically significant circadian rhythm (p<0.01) in *GstD1* expression levels in heads of wild type CS flies with the peak expression at ZT 8 and the trough 12 hours later at ZT 20 (Fig. 7A). This differential expression was abolished in heads of both *per^01^* and *cyc^01^* mutants such that similar low levels of *GstD1* mRNA were detected at both ZT 8 and ZT 20 (Fig. 7B). Taken together, these data demonstrate that the circadian clock affects the expression of *GstD1*, as previously suggested by microarray studies [40]. Given that *GstD1* expression in *Drosophila* is induced via *Keap1/Nrf2* signaling [39], we also examined the transcriptional profiles of *cncC*, (the *Drosophila* homologue of mammalian *Nrf2* gene), and *Keap1* genes. We found no circadian rhythms in *cncC* or *keap1* mRNAs, nor was there any effect of *per* or *cyc* mutations on their mRNA expression levels (Figure S1).

## Discussion

This study advanced our understanding of the effects of circadian clocks on cellular homeostasis. We found that the circadian system regulates *de novo* synthesis of glutathione by direct transcriptional control of the genes encoding GCL subunits, as well as modulation of the activity of the GCL holoenzyme and hence, its end-point product, GSH. Given the conserved nature of the circadian clock and that many metabolites linked to redox show diurnal oscillations in mammals [21], [41] the molecular connections we established here between the circadian clock and GSH biosynthesis may be conserved across different phyla.

We show that GSH undergoes circadian fluctuations in *Drosophila* heads, reaching its highest levels in the morning. While diurnal GSH variations were previously reported in different mammalian organs, such as the liver [42], the underlying molecular mechanism was not elucidated. A critical finding of our study is that the generation of the GSH rhythm in *Drosophila* heads involves transcriptional regulation of genes that encode subunits comprising GCL, the first and rate limiting enzyme in glutathione production. Daily rhythms for both *Gclm and Gclc* mRNA were discerned in LD with peak expression in the early and late night, respectively. However, *Gclc* mRNA did not show significant fluctuations in DD, suggesting that the rhythm may have dampened or is modulated by LD. On the other hand, the expression of both genes was significantly altered in mutants with defects in the positive or negative arm of the clock loop. Namely, expression of *Gclc* and *Gclm* was lower at the expected peak in *cyc^01^* flies, which have a disrupted CLK/CYC complex, and higher at the expected trough in *per^01^* mutants lacking periodic repression of CLK/CYC activity. Thus, our functional genetic data suggest that *Gclc* and *Gclm* may be activated by the CLK/CYC complex. This conclusion is consistent with a recent genome-wide study suggesting that CLK/CYC binds chromatin in the vicinity of the *Gclc* and *Gclm* gene promoters in a time dependent manner [7]. Since CLK binding could not be unambiguously mapped because of its occurrence near transcription start sites of genes adjacent to *Gclc* and *Gclm* [7], we investigated the expression of these neighboring genes and found them to be non-rhythmic.

Because GSH biosynthesis is critical for cellular health, transcriptional regulation of *Gclc* and *Gclm* have been studied intensively in mammals [19]. These genes are known to be induced by oxidative stress and electrophiles through the binding of stress responsive transcription factors to AP-1 and electrophile response elements [43], [44]. Analysis of DNA regulatory regions revealed the presence of such consensus motifs in the *Drosophila Gclc* and *Gclm* promoters (S. Radyuk, unpublished). In mammals, *Gclc* is induced via *Keap1/Nrf2* signaling; thus we examined the transcriptional profiles of *cncC*, (a *Drosophila* homologue of mammalian *Nrf2* gene), and *Keap1*. We did not detect a circadian rhythm for either *cncC* or *Keap1* mRNAs, nor was there any effect of *per* or *cyc* mutations on their mRNA expression levels. However, it remains possible that post-transcriptional modification of these factors could be involved in the temporal modulation of *Gclc* and *Gclm* expression.

In contrast to the robust rhythmic expression of *Gclc* and *Gclm* mRNAs, the protein levels of GCLc did not appear rhythmic, while variations in the GCLm protein levels were significant but modest. Nevertheless, we detected a significant daily rhythm in GCL activity. There are many factors that affect the GCL enzyme activity, among which are the relative proportions of GCLc and GCLm proteins, their posttranslational modifications, as well as substrate levels [19], [35], [36], [45]. The GCLc/GCLm ratio showed a trend toward a daily rhythm, which could contribute to the observed changes in GCL enzyme activity. Previous *in vitro* studies suggested that GCL activity is inhibited by GSH in both mammals and *Drosophila* [35]–[37]. Remarkably, our *in vivo* study determined that GCL enzymatic activity and GSH levels oscillate in phase with each other such that the highest levels of GCL activity overlap with elevated GSH in the early morning. Thus, our *in vivo* study may uncover new layers of physiological regulation involving these key redox components.

Rhythm in GSH biosynthesis could be important for many aspects of clock-controlled cellular homeostasis since this prevalent endogenous compound acts as a major antioxidant, regulates activity of detoxification enzymes, and mediates redox-sensitive signaling. GSH functions in the central nervous system also include maintenance of neurotransmitters, and membrane protection [46], [47]. Our previous study suggested that ROS and oxidative damage levels fluctuate in heads of wild type flies [15] raising a possibility that GSH rhythms may be linked to these phenotypes. However, the mechanism remains to be elucidated as GSH does not directly react with peroxides. The removal of hydrogen peroxide and other peroxides occurs in high-turnover reactions catalyzed by glutathione peroxidases and peroxiredoxins [48]–[50]. Interestingly, some of these enzymes display circadian oxidation-reduction cycles in model organisms across phyla, including *Drosophila* [51].

An important function of GSH is in phase II detoxification, in which GSH is conjugated with xenobiotics and metabolic by-products in reactions catalyzed by glutathione S-transferases [52], [53]. We report here that mRNA levels of *GstD1*, a known antioxidant and detoxification response gene in *Drosophila* [38], [39], [54] is expressed rhythmically in heads of wild type flies. This is consistent with previous microarray-based analyses which suggested that *GstD1* and several other GSTs are expressed rhythmically in the adult *Drosophila* head [40], [55], [56]. Interestingly, *GstD1* expression peaks in mid-day, when GSH levels become significantly reduced (compare Fig. 1 and 7). Other GSTs also peak at this time [40], suggesting a scenario where GSH is depleted due to conjugation and then replenished later in the circadian cycle. It has been hypothesized that the clock may coordinate redox responses as part of a strategy to increase the potential for neutralization of toxins during the morning when flies are active [40]. In agreement with this view, we showed that the circadian clock regulates susceptibility to pesticides as well as expression of specific genes that control xenobiotic metabolism [12], [13].

Although a significant rhythm in the GSH levels and GCL activity was detected in flies with an intact clock, the rhythm was not apparent in clock mutants. Instead, both of these parameters remained relatively elevated around the clock, more similar to the peak rather than the trough levels of the control (Fig. 1B & 6B). It is conceivable that this enhanced constitutive GSH production may result in an imbalanced redox state, which in turn could compromise redox signaling leading to physiological deficits. Consistent with this inference is the observation of adverse effects on fly survivorship when GCLc was over-expressed ubiquitously, resulting in high levels of GSH production [29], [34], (S. Radyuk, unpublished observations). In other studies, we showed that accumulation of carbonylated proteins and peroxidated lipids is accelerated in *per^01^* flies relative to age-matched controls [16], and that *per^01^* mutants are more susceptible to neurodegeneration [17]. Taken together, these data suggest that daily fluctuations in GSH may promote the health of the nervous system more efficiently than if GSH is maintained at constitutively elevated levels. Another important point is that while *per^01^* exhibits constant high GSH levels, the expression of the GSH-conjugating enzyme *GstD1* is significantly reduced in this mutant. This suggests that dysregulation between GSH supply and utilization may occur in clock-deficient flies.

One important question that remains to be addressed is whether rhythms in GSH-biosynthesis are controlled cell-autonomously or systemically. The circadian system in fly heads consists of several clusters of central pacemaker neurons forming a circuit responsible for circadian rhythms of locomotor activity [57]. In addition, retinal photoreceptors, sensory neurons, glia, and other cells contain a molecular clock mechanism, which can function independently of the central pacemaker [6], [58]. Transcriptional rhythms that are detected in whole heads may be generated in peripheral oscillators. Nevertheless, at least some central pacemaker neurons appear to be among the cells showing transcriptional *Gclc* and *Gclm* rhythms, based on microarray analysis of isolated pacemaker cells [59]. While the range of cells displaying rhythmic GSH biosynthesis remains to be determined, it is likely to be broad. A recent genome-wide study suggests that circadian expression of *Gclc* may occur in isolated fly brains [25], and our data suggest that *Gclc* and *Gclm* expression is also rhythmic in fly bodies (Dani Long and Eileen Chow, unpublished).

What is the biological advantage of adding a circadian level of regulation to GSH biosynthesis? While excessive ROS levels are detrimental to cell function, some levels of ROS are necessary in the organism, as these molecules are responsible for essential processes including cell signaling cascades and immune response. Thus, GSH acts not only as an antioxidant, but also plays a critical role in a plethora of redox-sensitive cellular functions (reviewed in [49]). While over-expression of GCLc in *Drosophila* neuronal tissue, and thus increased GSH levels, correlated with protection against oxidative stress and extension of lifespan [34], [60], recent findings suggests that GSH may rather function via affecting specific metabolic and defense pathways [61]. An array of connections has been recently established between circadian clocks and metabolism in mammals [10], [41], [62] and in flies [63]. Our present study adds an important novel link to this array by demonstrating circadian control of glutathione, a compound that is critically involved in maintaining human health.

## Supporting Information

Figure S1 **No significant circadian rhythm was detected in (A)** ***cncC*** **and (B)** ***Keap1*** **mRNA levels over the circadian day in the heads of wild type CS males.** A 1-way ANOVA and Dunnett's post-test showed p>0.05. No significant difference was observed in (C) *cncC* or (D) *Keap1* mRNA levels at ZT 8 or ZT 20 between wild type (CS), *per^01^* and *cyc^01^* flies. Data were analyzed by a 2-way ANOVA and Dunnett's post-tests and p>0.05. (A–D) Data represent average values (± SEM) obtained from 3 independent bio-replicates and normalized to ZT 0 (A–B) or ZT 8 (C–D).
(PDF)

Supplementary Methods S1 **Validation of the GSH and γ-GC detection methods and improvement of GSH detection in fly heads.**
(DOCX)

Table S1 Summary of the forward and reverse sequences of PCR primers used for quantitative RT-PCR analysis in alphabetical order.
(PPTX)
