# Supplementary figures and images for: Circadian Regulation of Glutathione Levels and Biosynthesis in Drosophila melanogaster
Source: PLoS One. 2012 Nov 30;7(11):e50454. doi: 10.1371/journal.pone.0050454 (PMC3511579; doi:10.1371/journal.pone.0050454)

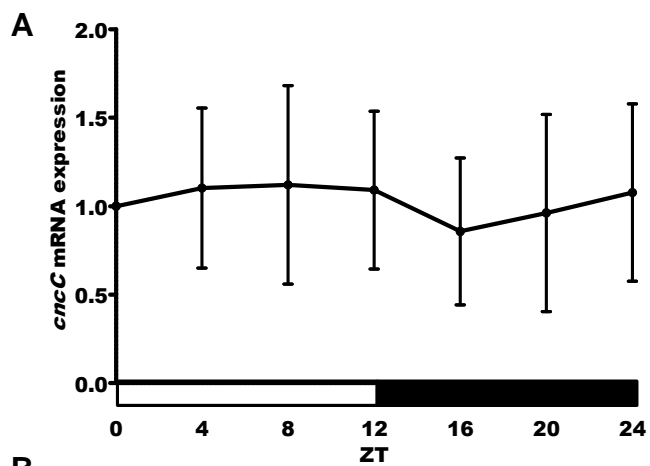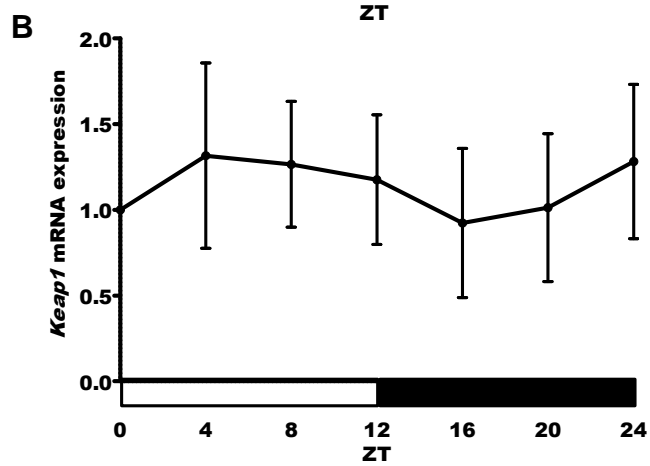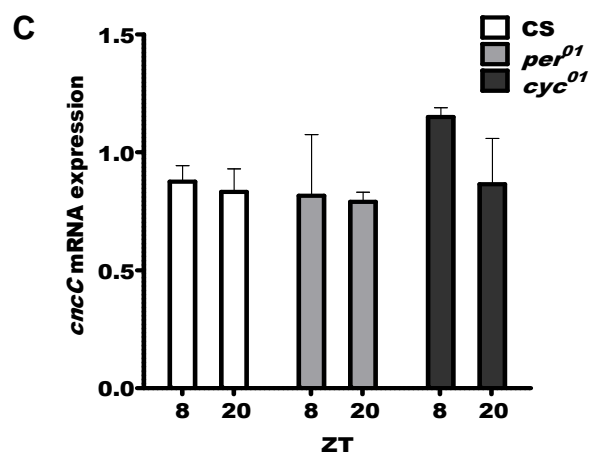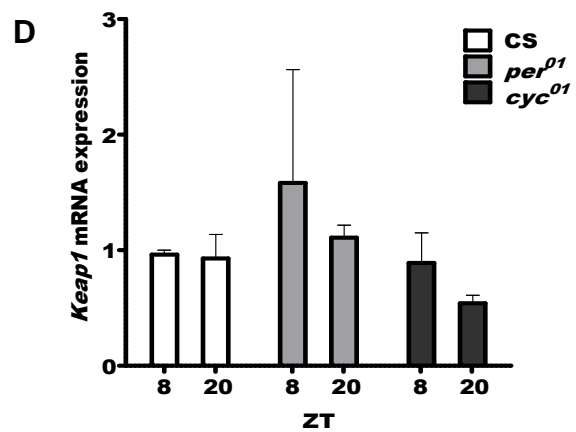

Supplement: Figure S1 — No significant circadian rhythm was detected in (A) cncC and (B) Keap1 mRNA levels over the circadian day in the heads of wild type CS males. A 1-way ANOVA and Dunnett's post-test showed p>0.05. No significant difference was observed in (C) cncC or (D) Keap1 mRNA levels at ZT 8 or ZT 20 between wild type (CS), per01 and cyc01 flies. Data were analyzed by a 2-way ANOVA and Dunnett's post-tests and p>0.05. (A–D) Data represent average values (± SEM) obtained from 3 independent bio-replicates and normalized to ZT 0 (A–B) or ZT 8 (C–D). (PDF) [file pone.0050454.s001.pdf]
