# Supplementary material for: Circadian Regulation of Glutathione Levels and Biosynthesis in Drosophila melanogaster
Source: PLoS One. 2012 Nov 30;7(11):e50454. doi: 10.1371/journal.pone.0050454 (PMC3511579; doi:10.1371/journal.pone.0050454)
Supplement: Supplementary Methods S1 — Validation of the GSH and γ-GC detection methods and improvement of GSH detection in fly heads. (DOCX) [file pone.0050454.s002.docx]

**Validation of the GSH and γ-GC detection methods**

The column efficiency was determined as N=5.545(RT/W_h/2_)^2^, where N is a separation efficiency (theoretical plates), RT is a retention time and W_h/2_ is a peak width at a half-height. The efficiency was 8370 for GSH, 9849 for γ-GC and 15469 for L-methionine.

The retention time and peak area repeatability was measured by 10 repetitive injections of 10 μM GSH, 2 μM γ-GC and 5 μM L-methionine standards. The coefficient of variation (CV) of peak area was 0.23, 0.34 and 0.41% for GSH, γ-GC and L-methionine standards, respectively. The CV of retention time was 0.18, 0.19 and 0.21% for GSH, γ-GC and L-methionine, respectively.

Concentration standards 0.1, 0.3, 1, 3, 10 and 30 μM of GSH, γ-GC and L-methionine were used to generate calibration curves, which were linear in this range (0.1-30 µM). Linear relationship between the peak area (y) and concentration standard (x) was: y = 3.006x + 0.002025 for GSH; y = 2.817x – 0.000645 for γ-GC; y = 1.465x + 0.000125 for L-methionine. Coefficient of determination was >0.999.

The limit of detection for GSH and γ-GC was 30 fmol per 10 µl injection at a signal-to-noise ratio of 3.

The recovery of GSH in the extract spiked with 3 and 10 μM of the GSH standard was 98.6% and 101.3%, respectively.

The reaction mixture for the GCL assay contained 5 mM cysteine​​, which produced a high signal and caused variations in the electrode responsiveness at +750 mV. Precision of γ-GC detection was improved with introduction of L-methionine as an internal standard. The calibration curve for γ-GC detection in the GCL activity assay reaction mix was generated using 1, 2, 3 and 4 μM of the γ-GC standards and 5 μM L-methionine as an internal standard and was linear in this range. It resulted in equation y=0.385x –0.006227, where y is a ratio of γ-GC standard area to internal standard (L methionine) area, and x is an γ-GC standard concentration. Coefficient of determination was =0.9997.

The intra-assay precision of γ-GC detection was determined in a single run of 6 replicates of the same reaction mixture. The CV was 3%.

The inter-assay precision of γ-GC detection was determined by analyzing the same reaction mixture on 3 different days over the week. The CV was 4.8%.

**Improvement of GSH detection in fly heads**

Extracts from different tissues have different constituents and may contain analytes co-eluting with GSH and thus compromising analysis, Alkylated GSH is not electrochemically active. After treatment of fly head extracts with alkylating agent, NEM, we found that the area of the peak where GSH is normally eluted contains 3-4% of other analytes. Consequently, the contribution of contaminating analytes was subtracted to obtain accurate GSH values.

The intra-assay precision of GSH detection was determined in a single run of 6 replicates of the same extract (NEM treated and untreated). The CV was 1.15%.

The inter-assay precision of GSH detection was determined by analyzing the same extract on 3 different days over the week. The CV was 3.9%.
