# Supplementary material for: Circadian Regulation of Glutathione Levels and Biosynthesis in Drosophila melanogaster
Source: PLoS One. 2012 Nov 30;7(11):e50454. doi: 10.1371/journal.pone.0050454 (PMC3511579; doi:10.1371/journal.pone.0050454)
Supplement: Table S1 — Summary of the forward and reverse sequences of PCR primers used for quantitative RT-PCR analysis in alphabetical order. (PPTX) [file pone.0050454.s003.pptx]

## Slide 1
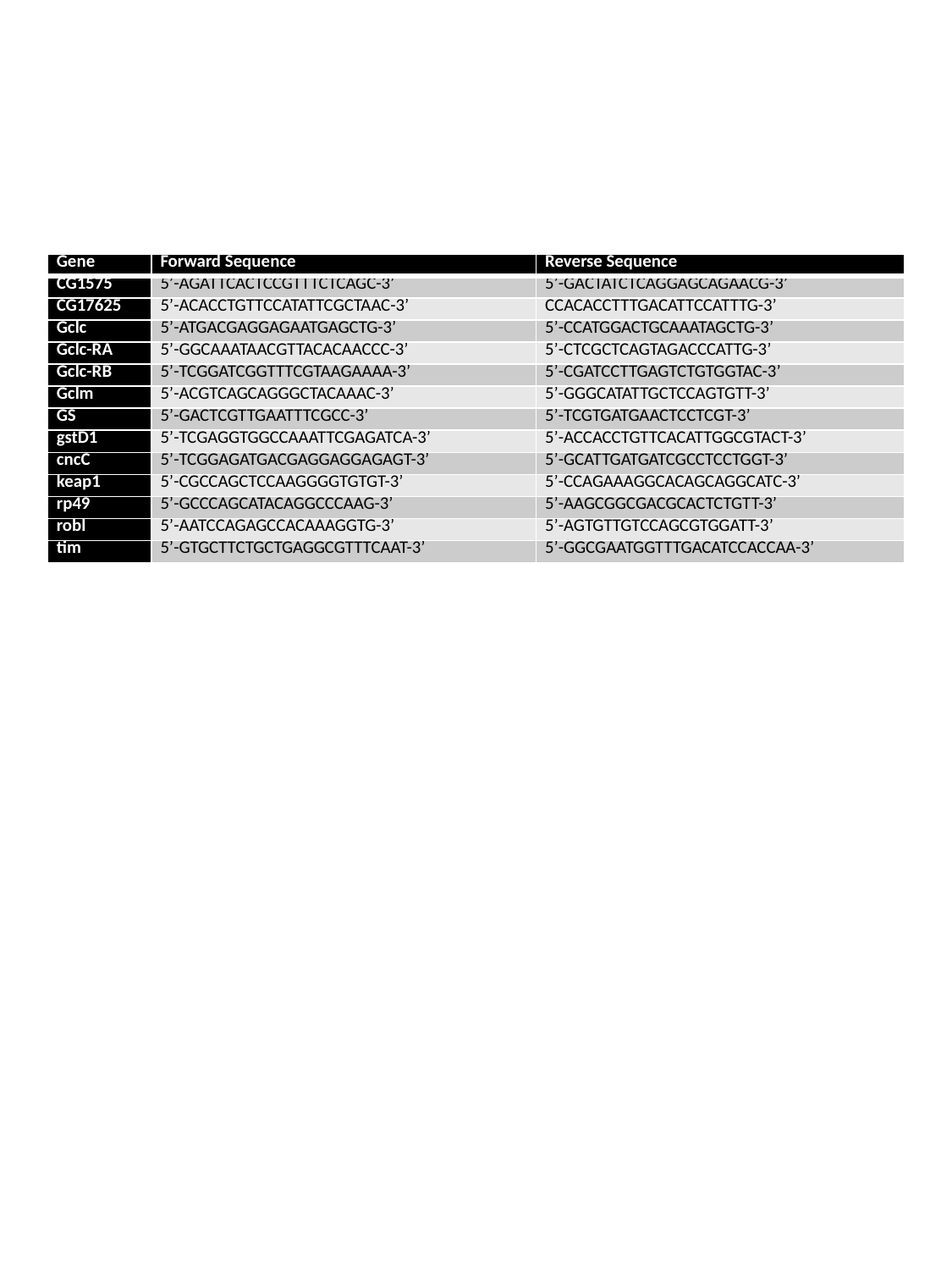

| Gene | Forward Sequence | Reverse Sequence |
| --- | --- | --- |
| CG1575 | 5’-AGATTCACTCCGTTTCTCAGC-3’ | 5’-GACTATCTCAGGAGCAGAACG-3’ |
| CG17625 | 5’-ACACCTGTTCCATATTCGCTAAC-3’ | CCACACCTTTGACATTCCATTTG-3’ |
| Gclc | 5’-ATGACGAGGAGAATGAGCTG-3’ | 5’-CCATGGACTGCAAATAGCTG-3’ |
| Gclc-RA | 5’-GGCAAATAACGTTACACAACCC-3’ | 5’-CTCGCTCAGTAGACCCATTG-3’ |
| Gclc-RB | 5’-TCGGATCGGTTTCGTAAGAAAA-3’ | 5’-CGATCCTTGAGTCTGTGGTAC-3’ |
| Gclm | 5’-ACGTCAGCAGGGCTACAAAC-3’ | 5’-GGGCATATTGCTCCAGTGTT-3’ |
| GS | 5’-GACTCGTTGAATTTCGCC-3’ | 5’-TCGTGATGAACTCCTCGT-3’ |
| gstD1 | 5’-TCGAGGTGGCCAAATTCGAGATCA-3’ | 5’-ACCACCTGTTCACATTGGCGTACT-3’ |
| cncC | 5’-TCGGAGATGACGAGGAGGAGAGT-3’ | 5’-GCATTGATGATCGCCTCCTGGT-3’ |
| keap1 | 5’-CGCCAGCTCCAAGGGGTGTGT-3’ | 5’-CCAGAAAGGCACAGCAGGCATC-3’ |
| rp49 | 5’-GCCCAGCATACAGGCCCAAG-3’ | 5’-AAGCGGCGACGCACTCTGTT-3’ |
| robl | 5’-AATCCAGAGCCACAAAGGTG-3’ | 5’-AGTGTTGTCCAGCGTGGATT-3’ |
| tim | 5’-GTGCTTCTGCTGAGGCGTTTCAAT-3’ | 5’-GGCGAATGGTTTGACATCCACCAA-3’ |
